# A Case Report of Lateral Subtalar Dislocation: Emergency Medicine Assessment, Management and Disposition

**DOI:** 10.21980/J8SS8P

**Published:** 2024-07-31

**Authors:** Alexander Maybury, Taylor Isenberg

**Affiliations:** * Morristown Medical Center, Department of Emergency Medicine, Morristown, NJ

## Abstract

Subtalar dislocations of the talonavicular and talocalcaneal joints are rare, accounting for approximately one percent of all dislocations.^1^ These dislocations are typically the result of a high energy mechanism and present a challenge during reduction attempts. We present the case of a male in his early 20’s who presented to the emergency department after a motorcycle accident with right foot and ankle pain and obvious deformity. Emergent X-ray and immediate attempt at reduction are of utmost importance with these dislocations. After multiple failed attempts at reduction in the ED, this patient was taken to the operating room for an open reduction with podiatry. This case report reviews the pathophysiology and management of this rare injury including nerve blocks and reduction techniques.

**Topics:**

Subtalar dislocation, trauma, podiatry, joint reduction, nerve blocks, local anesthesia.

[Fig f1-9-3-v5] [Fig f2-9-3-v5] [Fig f3-9-3-v5]

**Figure f1-9-3-v5:**
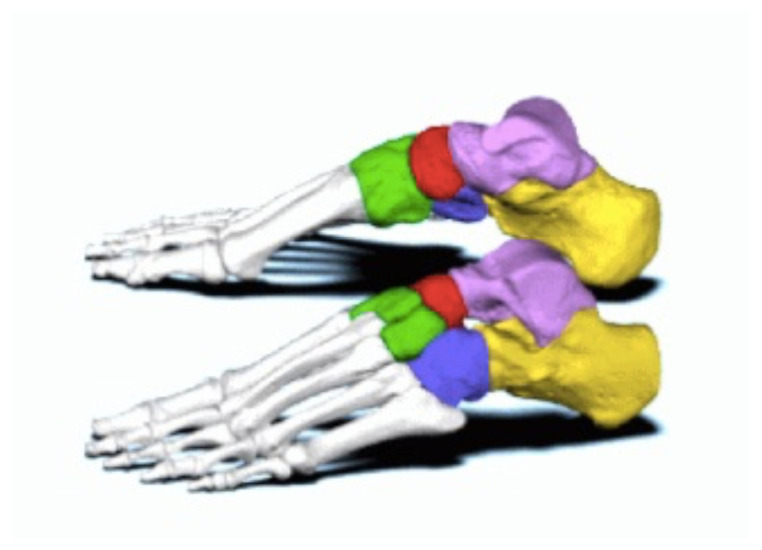


**Figure f2-9-3-v5:**
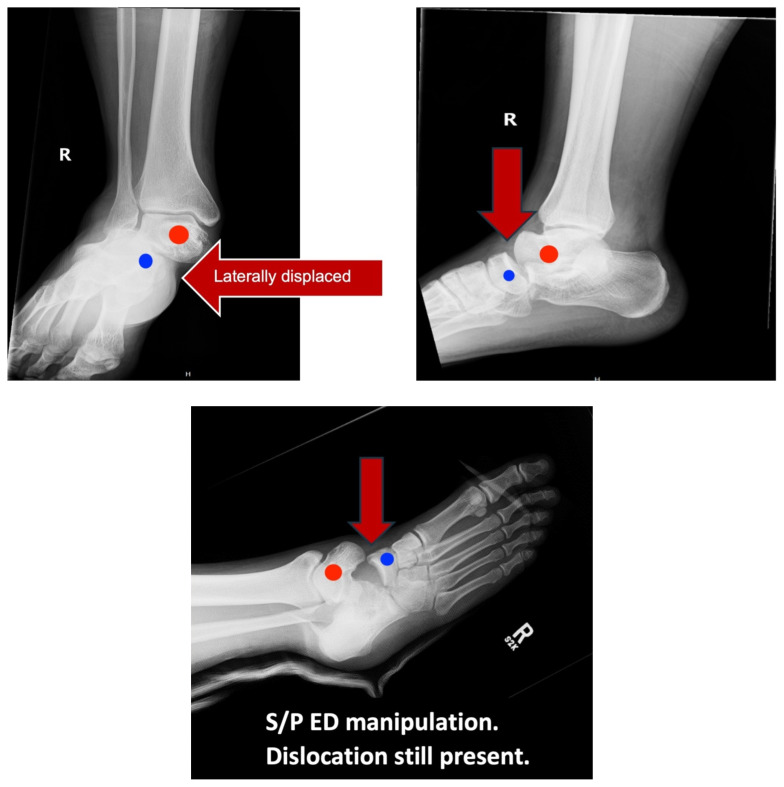


**Figure f3-9-3-v5:**
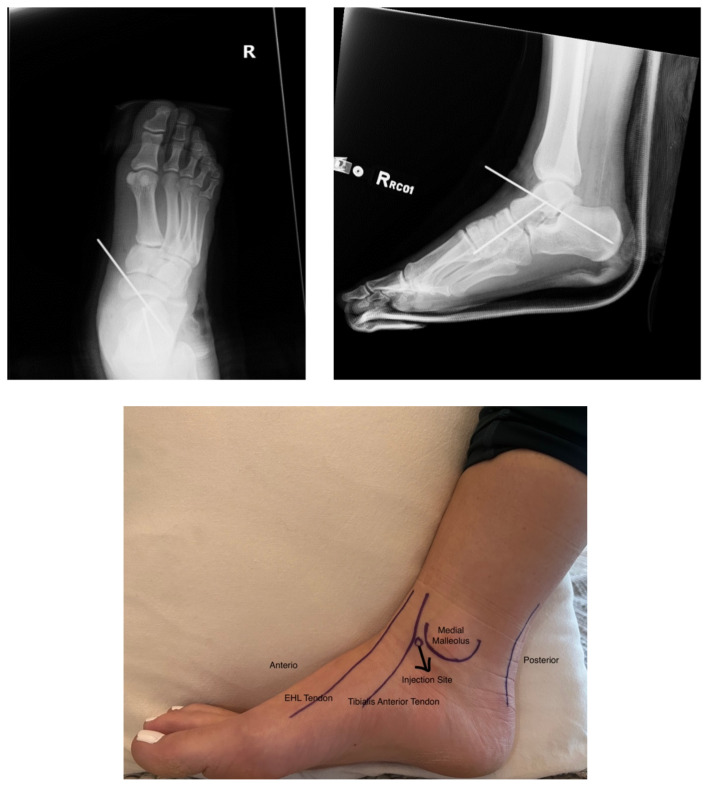


## Brief introduction

Subtalar dislocations are rare and make up approximately one percent of all joint dislocations.^1^ They are most frequently seen in young to middle-aged males following a high energy traumatic mechanism, such as sports injuries and motor vehicle collisions. These dislocations most commonly occur medially, but have been described laterally, posteriorly and anteriorly,^2^ Additionally, they can be both open and closed. Prompt neurovascular assessments are critical because injury to the tenuous blood supply of the subtalar region may lead to osteonecrosis and long-term disability.^3^ Upon patient presentation, if a subtalar dislocation is suspected, associated with neurovascular compromise, reduction attempts should not be delayed secondary to imaging studies. It is also important to expedite orthopedic or podiatric consultation because up to 32% of these dislocations require open reduction.^1^ Subtalar joint dislocations are rare; however, it is of the utmost importance for an EM physician to either attempt reduction as soon as possible or emergently consult surgical colleagues. These dislocations involve the hindfoot, with loss of the articular relationship between the talonavicular and talocalcaneal joint, and typically result after high energy trauma.^4^

## Presenting concerns and clinical findings

A male in his early 20’s with no significant past medical history presented to the emergency department with isolated right lower extremity pain after a motorcycle accident. The patient reported that his bike rolled on his leg during the accident, resulting in forceful eversion of his ankle. He was unable to move his right foot and had decreased sensation. Physical exam revealed a closed deformity to the right medial ankle and mid-foot with decreased distal capillary refill and weak posterior tibial pulse.

## Significant findings

In a lateral subtalar dislocation, the navicular bone (red bone in 3D anatomy image) and the calcaneus (yellow bone in 3D anatomy image) dislocate laterally in relation to the talus (lavender bone in 3D anatomy image).^5^ Plain film oblique and lateral X-rays demonstrate the initial dislocation (talus in red, navicular in blue). It is clear in the initial lateral view that there is loss of the talar/navicular articulation (noted by red arrow). The anterior-posterior x-ray is more challenging to discern the anatomy; however, the talus (red dot) is laterally displaced in comparison to the navicular (blue dot). The post reduction film shows persistence of the dislocation despite attempted reduction by the emergency medicine physician, thus indicating a failed attempt and the necessity of operative repair. Post-operative X-rays reveal the post operative reduction with instrumentation installed.

## Patient course

In the ED, an intraarticular/hematoma block with lidocaine was administered and closed reduction was attempted but was unsuccessful. Podiatry was emergently consulted and after further unsuccessful reduction attempts, the patient was taken to the operating room two hours later. The podiatry team attempted closed reduction under general anesthesia, but the injury required open reduction with two pins to stabilize the talonavicular joint. The patient was discharged two days post operatively. At three-month follow up, patient has resumed normal physical activity with minimal restriction.

## Discussion

Subtalar dislocations can be attributed to the strong ligaments connecting the talocalcaneal joint, which are broken up into intrinsic and extrinsic ligaments.^5^ Subtalar dislocations can be subdivided into classifications including medial (most common), lateral, anterior, posterior and total dislocations (least common).^6^

The diagnosis is typically made radiographically because the initial presentation can look very similar to a trimalleolar fracture or other ankle dislocations. In a lateral dislocation, the patient will present with the lower extremity in a pronated position and is more likely to result in bony exposure.^7^ As discussed above, radiographic findings include loss of the talarnavicular articulation as well as the talar head being inferior to the navicular on the lateral view of the x-ray. CT scans do not have a role in the initial diagnosis or presentation; however, they are useful in the post-reduction period to identify subtalar debris or other associated injuries.

Lateral subtalar dislocations are more challenging to reduce secondary to entrapment of soft tissue structures and/or bone fragments.^8^ The recommended reduction technique is flexion of the knee to 90 degrees and flexion of the hip while providing counter-traction, placing the ankle in plantar flexion. Next, the calcaneus is grasped with the dominant hand, and the dorsum of the foot is grasped with the opposite hand, placing a thumb over the navicular. Subsequently, the hindfoot is distracted, and direct pressure is applied to the talar head while hyper-everting the foot. An attempt is made to exaggerate the dislocation, followed by distraction, then inversion of the dislocation to achieve an adequate reduction.^9^ One study with 388 patients saw that external reduction was successful in 264 patients (68%) while 124 patients required open surgical intervention.^3^

There are many ways to achieve reduction in the emergency department including intra-articular blocks as well as sedation with opioid and narcotics. Anesthesia with an intra-articular block is not well studied in ankle dislocations; however, several small studies have shown their effectiveness. 10 For the intraarticular injection, the patient should be supine, and the affected ankle should be prepared with betadine (povidone-iodine solution). A 20-gauge needle is inserted into the medial aspect of the ankle joint, medial to the tibialis anterior tendon, with use of sterile technique.^10^ Once the joint is successfully penetrated, a hematoma should be aspirated, confirming appropriate placement of the needle, and the joint should be injected with about 10 mL of 1% lidocaine without epinephrine.^10^ The proper location of the joint injection is about one centimeter anterior to the medial aspect of the medial malleolus (see attached photo). Common procedural sedation medication such as propofol, ketamine or versed can be used to adequately reduce the joint either in conjunction with or instead of the above intra-articular block.

The tarsal body has a diffuse blood supply with rich anastomosis. The tarsal canal supplies a majority of the talar body while the deltoid artery supplies the medial third.^11^ In subtalar dislocations, the posterior tibial artery can become compressed, and since this artery supplies the tarsal canal, subsequently decreases perfusion to the body of the talus. During reduction attempts the deltoid artery can become compromised, causing complete lack of blood flow to the talar body, thus causing avascular necrosis. It is important to perform frequent neurovascular checks, because about 70% of lateral dislocations are associated with neurovascular injuries.^12^ In addition to avascular necrosis, another potential long-term complication is injury to the posterior tibialis tendon; injury to this tendon decreases the arch of the foot and results in difficulty ambulating.^8^

Successfully reduced subtalar dislocation can be discharged from the ED with a short posterior splint and the instruction to avoid weight bearing for four to six weeks. If the joint cannot be successfully reduced following an attempt at closed reduction, open reduction and internal fixation will be necessary. Common complications following these injuries are post traumatic arthritis and unsteadiness when ambulating on uneven ground.

## Supplementary Information




















