# Supplementary figures and images for: A Case Report of Lateral Subtalar Dislocation: Emergency Medicine Assessment, Management and Disposition
Source: J Educ Teach Emerg Med. 2024 Jul 31;9(3):V5–9. doi: 10.21980/J8SS8P (PMC11312880; doi:10.21980/J8SS8P)

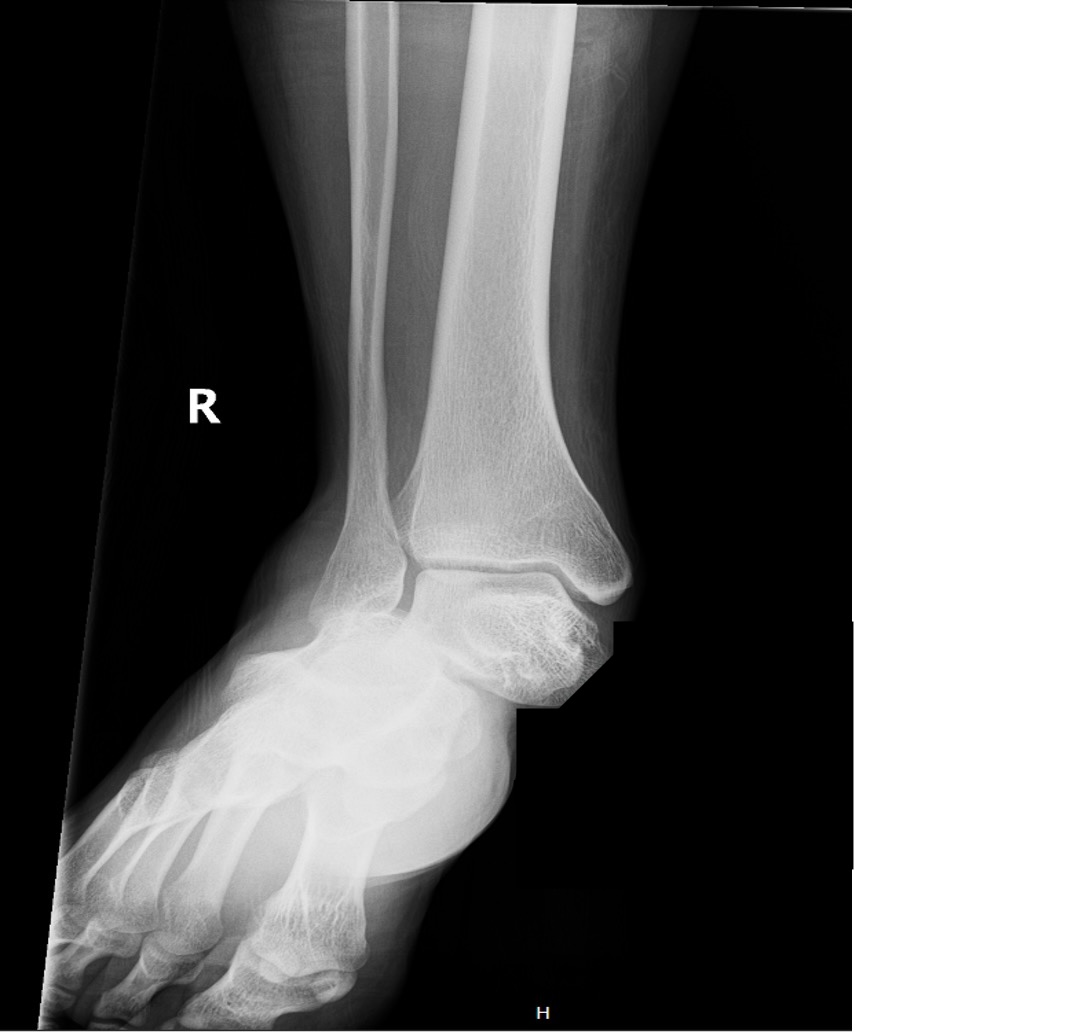

Supplement: Supplementary file 1 [file 9-3-V5-Supp1.jpg]

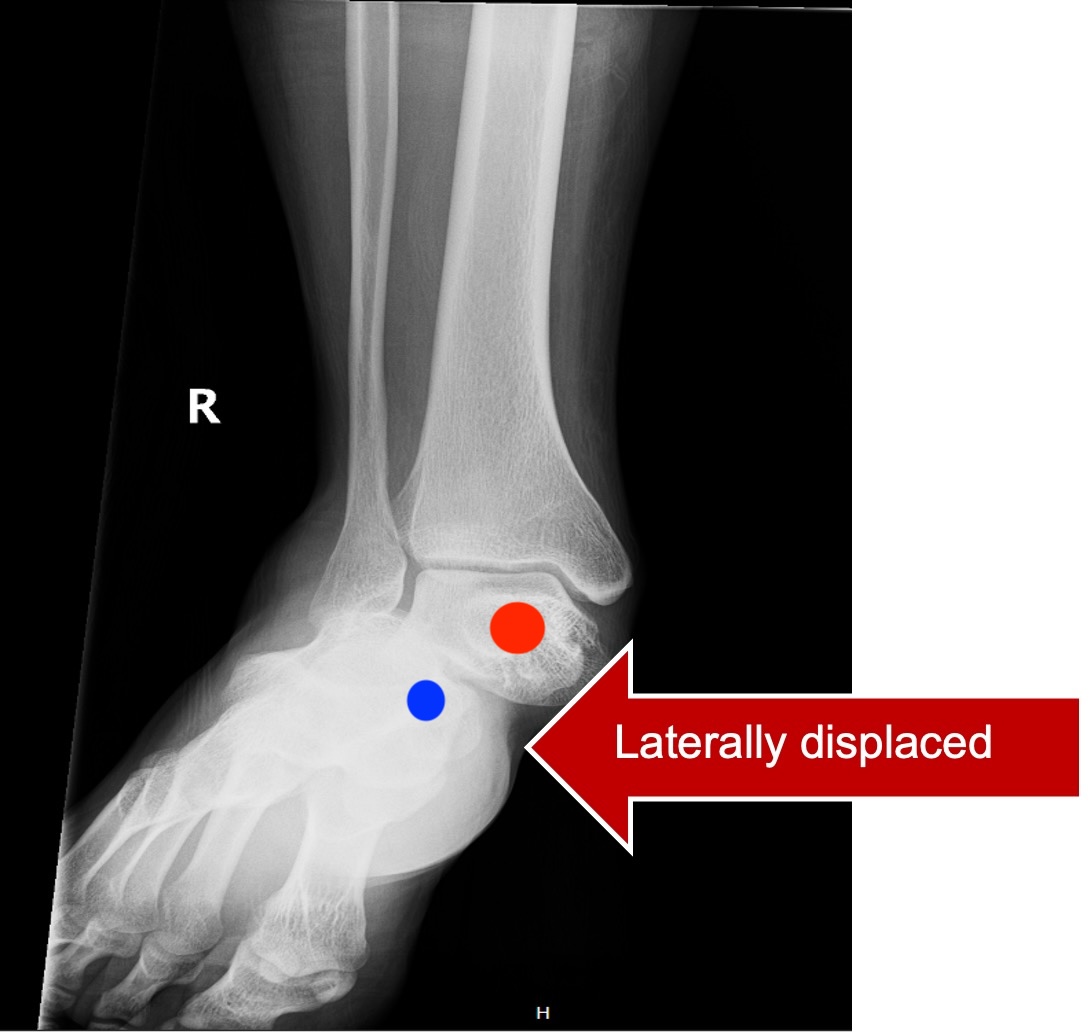

Supplement: Supplementary file 2 [file 9-3-V5-Supp2.jpg]

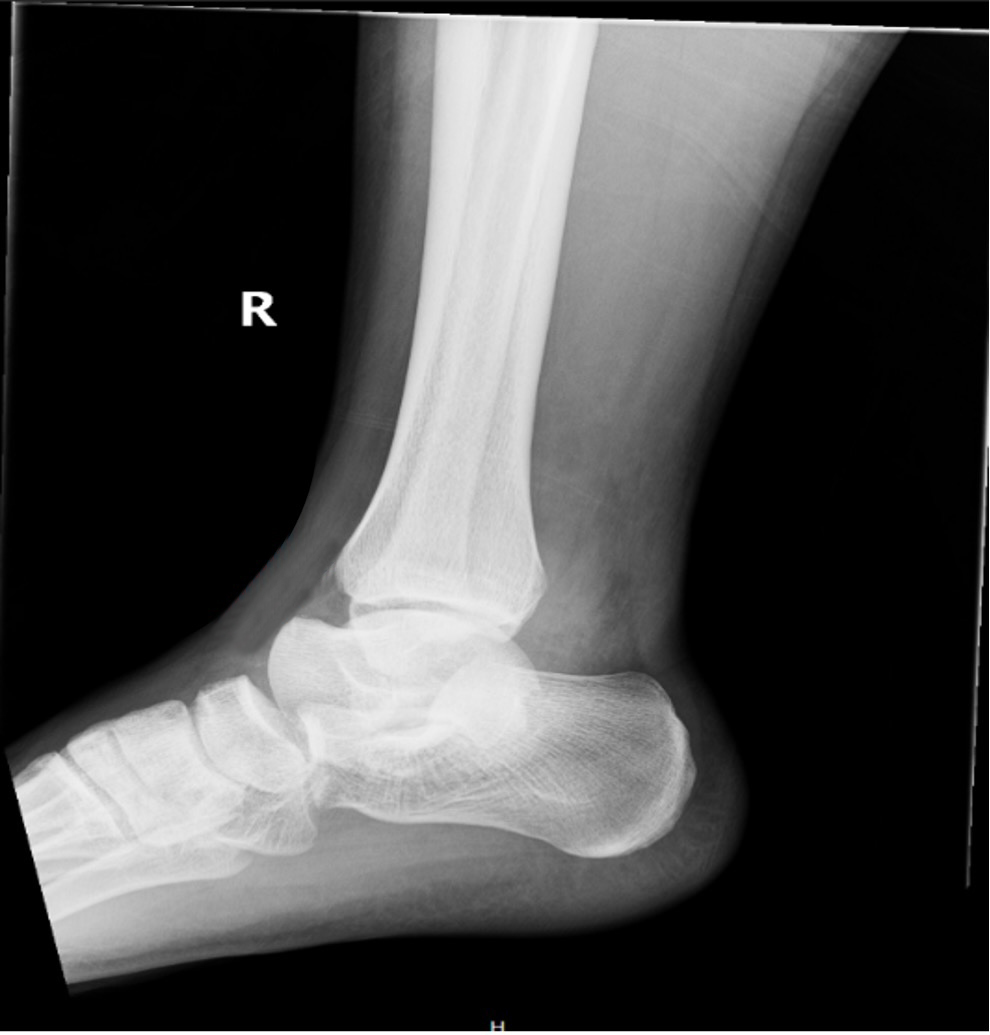

Supplement: Supplementary file 3 [file 9-3-V5-Supp3.jpg]

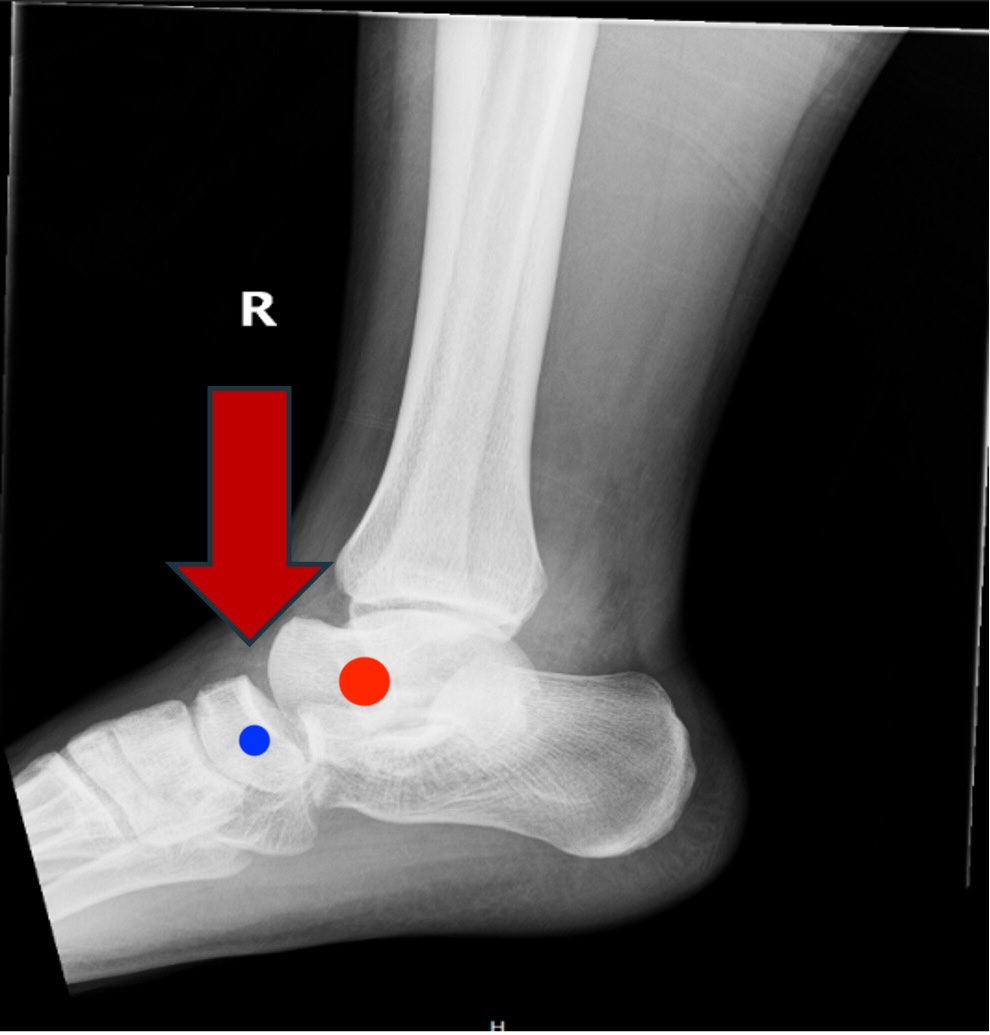

Supplement: Supplementary file 4 [file 9-3-V5-Supp4.jpg]

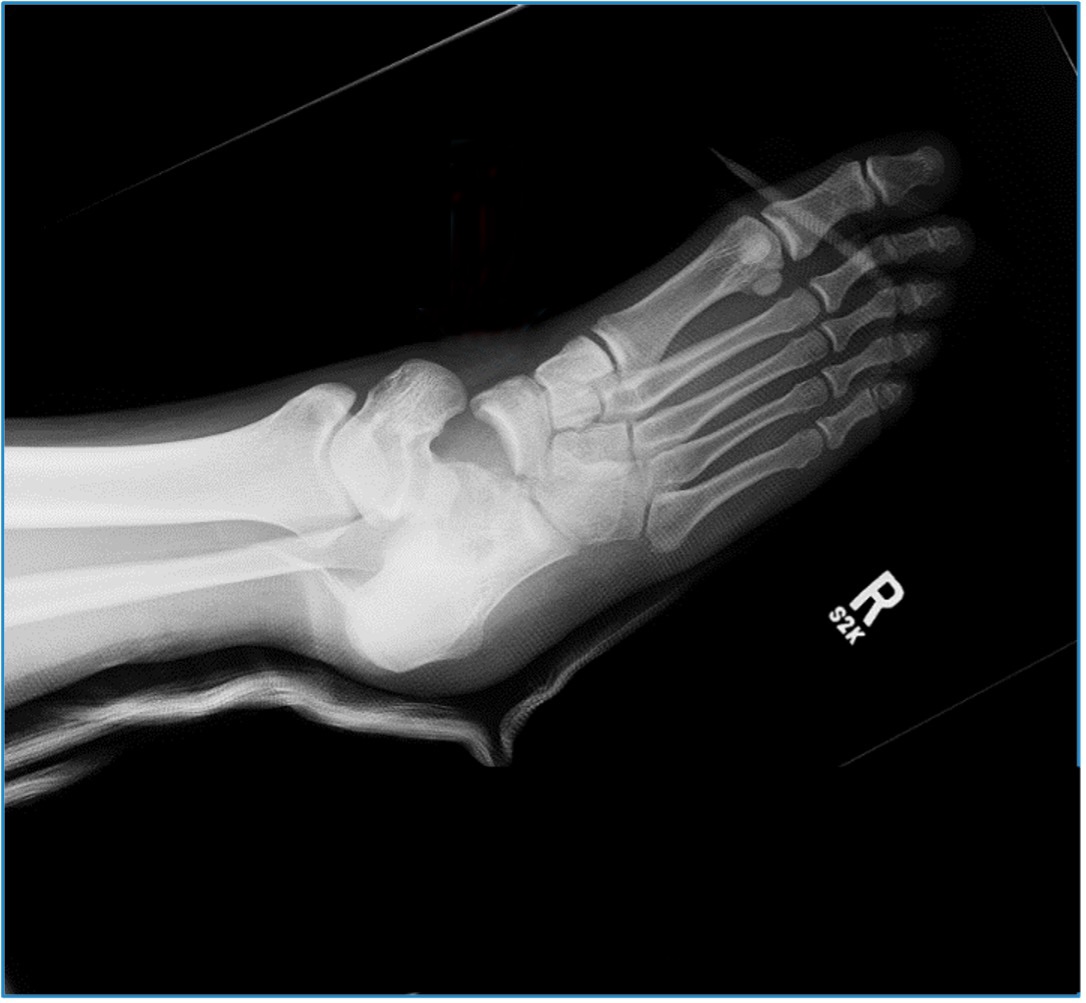

Supplement: Supplementary file 5 [file 9-3-V5-Supp5.jpg]

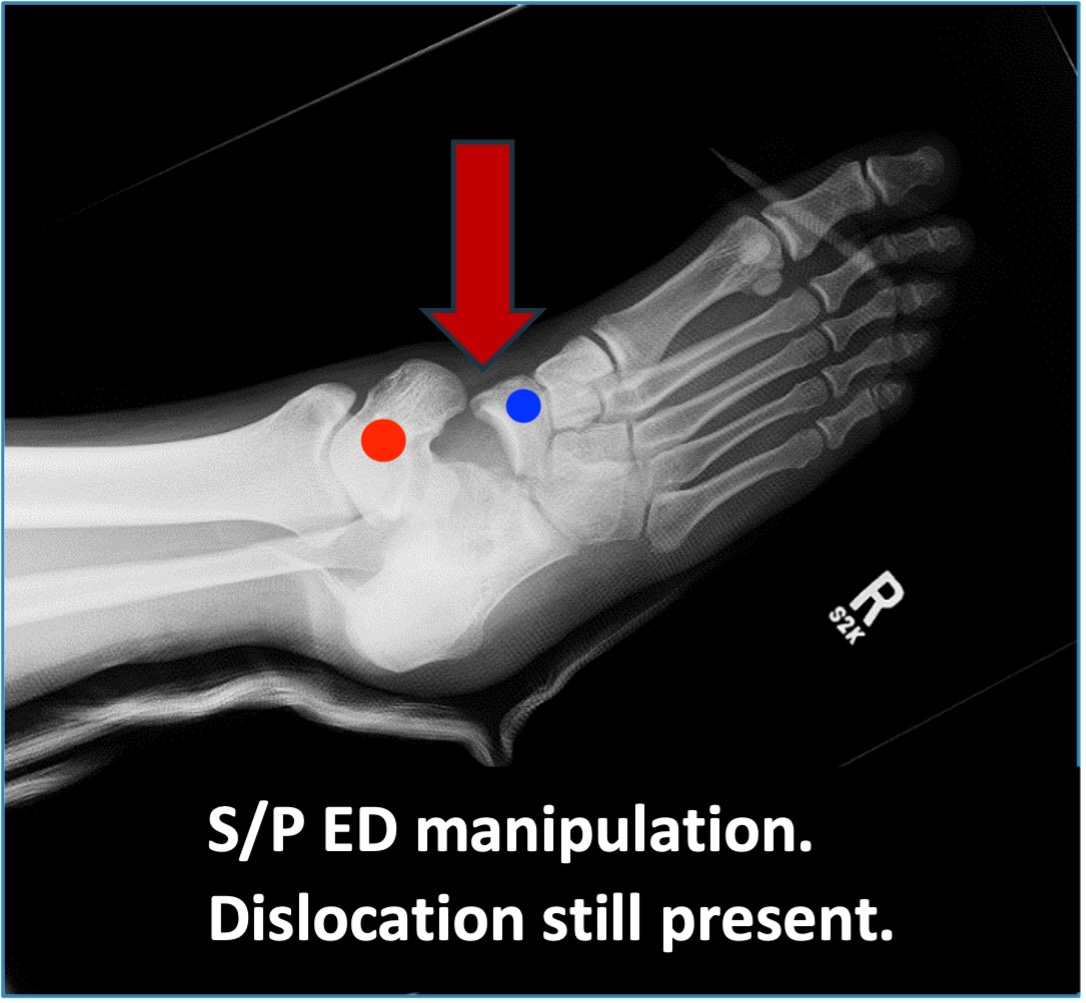

Supplement: Supplementary file 6 [file 9-3-V5-Supp6.jpg]

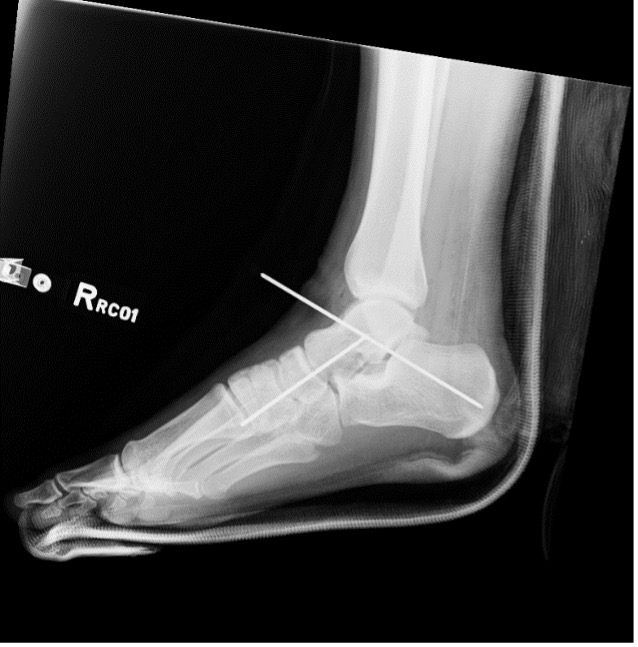

Supplement: Supplementary file 7 [file 9-3-V5-Supp7.jpg]

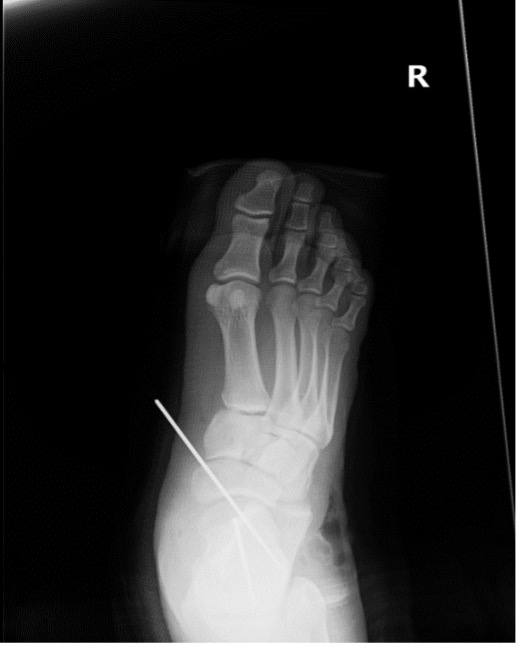

Supplement: Supplementary file 8 [file 9-3-V5-Supp8.jpg]

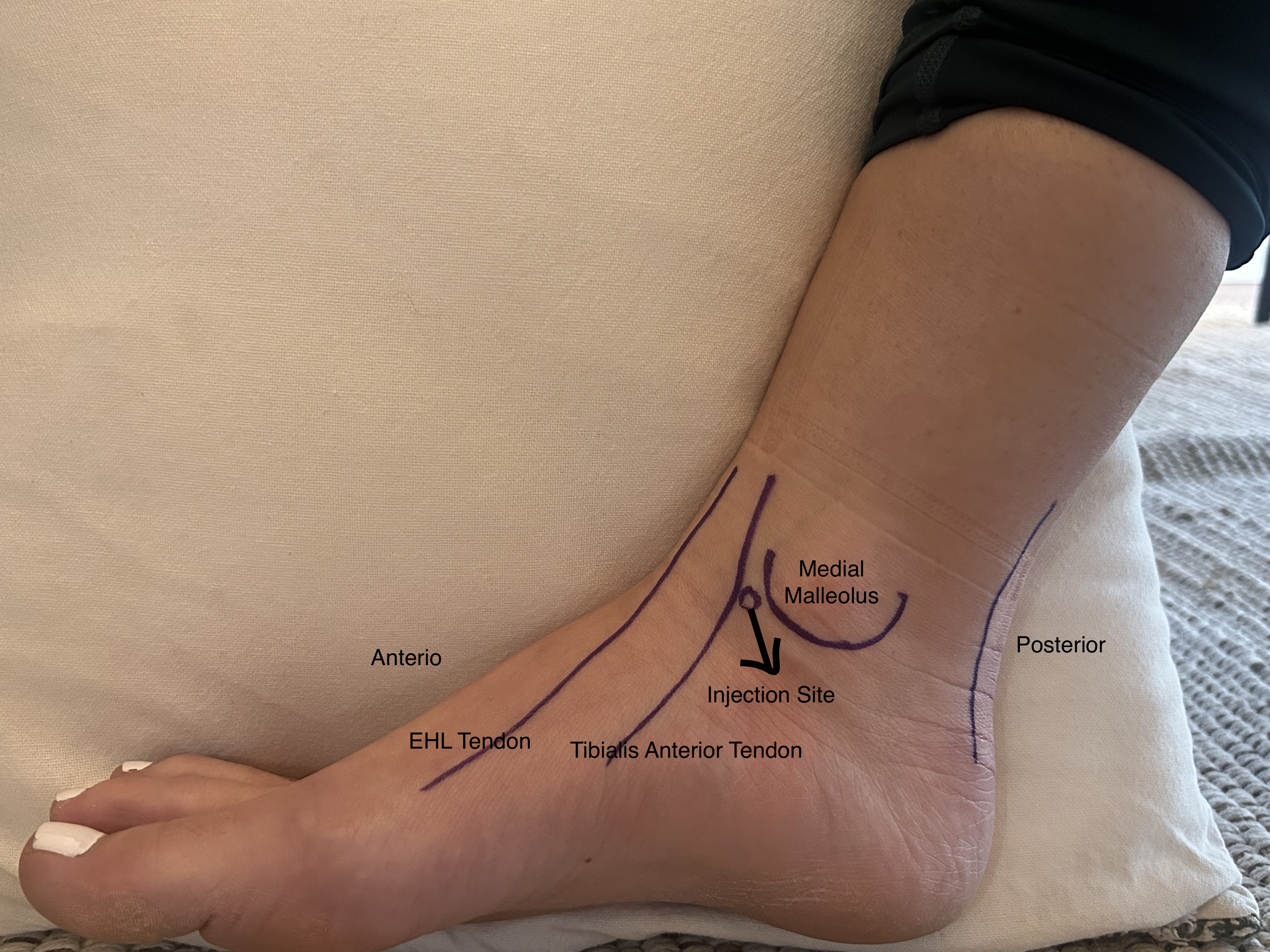

Supplement: Supplementary file 9 [file 9-3-V5-Supp9.jpg]

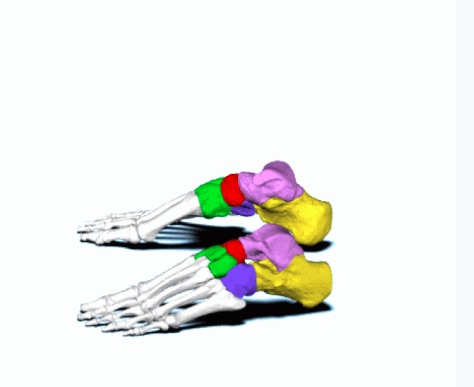

Supplement: Supplementary file 10 [file 9-3-V5-Supp10.jpg]
